# Temperature effects on development and fecundity of *Brachmia macroscopa* (Lepidoptera: Gelechiidae)

**DOI:** 10.1371/journal.pone.0173065

**Published:** 2017-03-02

**Authors:** Li Ma, Xing Wang, Yan Liu, Ming-Zhu Su, Guo-Hua Huang

**Affiliations:** 1 Hunan Provincial Key Laboratory for Biology and Control of Plant Diseases and Insect Pests, Changsha, China; 2 College of Plant Protection, Hunan Agricultural University, Changsha, China; Universita degli Studi della Basilicata, ITALY

## Abstract

The current study investigated the impacts of temperature on the development and reproductivity of the sweet potato leaf folder, *Brachmia macroscopa* (Lepidoptera: Gelechiidae), in sweet potato leaves under laboratory conditions. We determined developmental time of *B*. *macroscopa* larval, pupal, and pre-adult stage at different temperatures. Male and female longevity, male and female lifespan, mortality of immature stages, oviposition period of *B*. *macroscopa* were also investigated under six constant temperatures (21°C, 24°C, 27°C, 30°C, 33°C, 36°C), based on age-stage, two-sex life tables. The results revealed that eggs in 36°C were unable to hatch. At temperatures between 21°C -33°C, the duration of the pre-adult period, as well as the adult lifespan both for males and females, were shortened by increasing temperatures. The lowest larval mortality rate (15.33%) occurred at 27°C. The age-stage-specific fecundity rates with the greatest number were, in order, 30°C, 27°C, 21°C, 24°C and 33°C. The results show that *B*. *macroscopa* population levels could reach highest at the temperature of 27℃.

## Introduction

The sweet potato leaf folder, *Brachmia macroscopa* Meyrick (Lepidoptera: Gelechiidae), is one of the most destructive pests on *Dioscoreae sculenta*, *Ipomoea aquatic*, *Calystegia sepium*, *C*. *japonica* and many other crops belonging in the Convolvulaceae. It is widely distributed in Europe, Russia, Caucasus, the Transcaucasian region, West Kazakhstan, central Asia, Korea, Japan, China, and northern India [1]. Larval damage to the host plant leaves results in complete loss of the mesophyll layer, leaving only the transparent leaf epidermis. The extent of damage may be severe enough to disrupt the host’s ability to photosynthesize, causing withering of the affected leaves, and in severe cases ultimately lead to death of the host plant. Serious infestations may cause high host mortality and result in serious economic loss.

The biological responses of *B*. *macroscopa* larval, pupal and adult stages to different temperatures as well as a brief discussion of some of the pests’ thermal requirements have previously been reported [2,3]. To date, however, none of the previous studies have included quantitative life table data such as the many useful parameters that are obtainable from calculating two-sex life table parameters, including net reproductive rate (*R*_*0*_), gross reproductive rate (*GRR*), intrinsic rate of increase (*rm*), finite rete of increase (*λ*), and mean generation time (*T*), etc.

Life tables are widely accepted as a powerful and necessary tool for analyzing and understanding the effects abiotic and biotic factors such as temperature have on the growth, survival, reproduction, and intrinsic rate of increase of insect populations [4]. Different methods for analyzing life table data needed in studies on population ecology have been reported [4–8]. Life tables have also been widely adopted in ecological studies involving insect populations, including insect mass-rearing techniques [9–11], timing of pest control procedures [12], studies on host preference and fitness of insects [13, 14], as well as ecological studies on the effects that environmental variables, including temperature, have on populations dynamics [15, 16]. Population studies based on life tables other than the age-stage, two-sex life table can result in the incorporation of errors since they do not take the variation in developmental rates among individuals and between sexes into consideration [9, 17]. The age-stage, two-sex life table, on the other hand, was designed to allow incorporating variations in pre-adult development time, which, in turn, produces precise survival and fecundity curves. Research on the demography of an insect pest species under different temperature conditions has often been regarded as a basis for developing an eco-friendly management strategy. The objective of this study was to gather additional data on the biological and ecological properties of *B*. *macroscopa*, by conducting a study of its demographics using the results obtained from the age-stage, two-sex life table parameters under different temperature conditions. The ultimate goal in this study would be to provide key data needed to make appropriate, efficient, and strategic decisions in devising an effective control program for the species.

## Materials and methods

### Insect culture

Larvae, pupae and adults of *B*. *macroscopa* were collected from experimental fields belonging to Hunan Agricultural University (Changsha, Hunan, China; 28°110’, 113°40’). Insects used in each of the temperature treatments had been bred in the laboratory for at least two generations. Larvae were provided with freshly cut sweet potato leaves as a food source. The leaves were changed daily with freshly cut leaves to maintain hygienic conditions and to provide fresh leaves for the larvae until they reached the pre-pupal stage. Pupae and adults were kept in an insect rearing cage containing a sweet potato and allowed to mate. The sweet potato plants were covered with a mesh net to serve as an oviposition substrate. Adult nutrients consisted of a cotton ball saturated with a 30% honey solution which was replaced daily.

### Developmental time

In order to obtain eggs of uniform age, 20 pairs of female and male *B*. *macroscopa* were placed in a separate rearing container that was covered with mesh net to provide ventilation. A potted sweet potato plant, covered with mesh net as before, had been placed inside the cage to serve as the oviposition substrate. One hundred and fifty eggs were collected daily and used to initiate every temperature treatment, and separate cultures were maintained at different constant temperatures of 21, 24, 27, 30, 33, and 36°C, in artificial climate chambers at 75±20% RH and a photoperiod of 10L: 14D. Fresh sweet potato leaves were provided daily for the neonate larvae. Since each larva was considered as a replicate, they were individually transferred, using a fine brush, to separate plastic petri dishes (9 cm in diameter and 1.5 cm in height). To maintain freshness, the leaf petioles of the detached leaves were wrapped in water-soaked cotton. New pupae were placed separately in glass tubes (2 cm in diameter and 10 cm in height) containing a moistened cotton ball at the base of the tube, and the tubes covered with gauze. Daily records were kept for each individual, including recorded data for each larval molt, pupation and adult emergence, as well as noting any mortality that occurred. The entire larval period, prepupal period and pupal period, i.e., the developmental time from egg to adult emergence, was defined as the length of the pre-adult stage. In addition, the mortality of all stage was also exhibited. The experiment was continued until the death of all individual insects.

### Oviposition period, fecundity and longevity

When the pupae emerged as adults, individual males and females that had emerged during the same 24 hr period were paired and the pair transferred to a plastic oviposition container (13 cm in diameter and 17 cm in height). Each of the oviposition chambers contained a small cotton wick soaked with 20% honey solution for adult nutrition, along with a small sweet potato plant grown in a disposable cup to allow for oviposition. The potted plants were replaced daily and the number of eggs produced during that 24 hr period were recorded.

Other parameters including the adult pre-oviposition period (APOP; the period of time between the emergence of an adult female and the on set of her first oviposition), the total pre-oviposition period (TPOP; the time interval from birth to the beginning of oviposition), the oviposition period, daily fecundity, and total fecundity (total number of eggs produced during an individual’s reproductive period) were obtained using the experimental data.

### Life table parameters

The age-stage, two-sex life table approach was used to analyze the raw life-history data for *B*. *macroscopa* [18, 19]. The age-stage -specific survival rate (*s*_*xj*_) (= the probability that an individuals would survive to age *x*_*j*_ and stage *j*) [the first stage is the egg stage, the second stage is the 1st larval stage, the third stage is the 2nd larval stage, the fourth stage is the 3rd larval stage, the fifth stage is the 4th larval stage, the sixth stage is the 5th-6th larval stage (since the *B*. *macroscopa* fed on different temperatures had 5th to 7th stages, we grouped the 5th, 6th and 7th larval stages as the sixth stage), the seventh stage is the pupal stage, the eighth stage is the adult stage] was evaluated. The age-stage-specific fecundity (*f*_*xj*_) (daily number of eggs laid by individual at age *x* and stage *j*), the age-specific fecundity (*m*_*x*_) [(daily number of eggs produced per female (this number is divided by all individuals’ males and females)], and the age specific survival rate (*l*_*x*_) (the probability that the newly oviposited egg will survive to age *x*), were calculated [20]. The intrinsic rate of increase (*r*) was calculated using the Eule-Lotka equation as $\sum_{x=0}^{\infty }e^{-r_{m}(x+1)}l_{x}m_{x}=1$ [21]. The gross reproductive rate (*GRR*) was calculated as $\mathrm{GRR}=\sum m_{x}$. The finite rate of increase (*λ*) was calculated as $e^{r_{m}}$. The net reproductive rate (*R*_*0*_) was measured as $\sum_{x=0}^{\infty }l_{x}m_{x}$. The mean generation time (*T*) -defined as the time that the population could increase to *R*_*0*_-fold of its population size as it approaches the stable stage distribution, was calculated as *T* = (ln*R*_*0*_)/*r*_*m*_. Finally, the means, standard errors and variances of the population parameters were estimated via the bootstrap technique [22], which is contained in the TWOSEX-MSChart program. Sigma plot 12.5 was used to create the graphs.

### Data analysis

The raw life-history data obtained for *B*. *macroscopa* in each of the temperature treatments were entered separately into Microsoft Excel 2013. The computer program, TWOSEX-MSChart for the age-stage two-sex life table analysis in VISUAL BASIC (version 6, service pack 6) for the Windows system available at http://140.120.197.173/Ecology/ Download/TWOSEX-MSChart.zip (National Chung Hsing University) and at http://nhsbig.inhs.uiuc.edu/wes/chi.html (Illinois Natural History Survey), was used for analysis of the raw data. The computer program greatly simplifies the otherwise lengthily procedure required for analysis of the large amount of data generated in life table studies. One-way analysis of variance (ANOVA) was adopted to analyze the duration data. The LSD test was used to detect significant differences in the statistical analysis among means at *P = 0*.*05*. Nonlinear regression was used to analyze the development rates with the temperatures (using SPSS 19.0)

## Results

### Development time, adult longevity and lifespan

**Fig 1:**
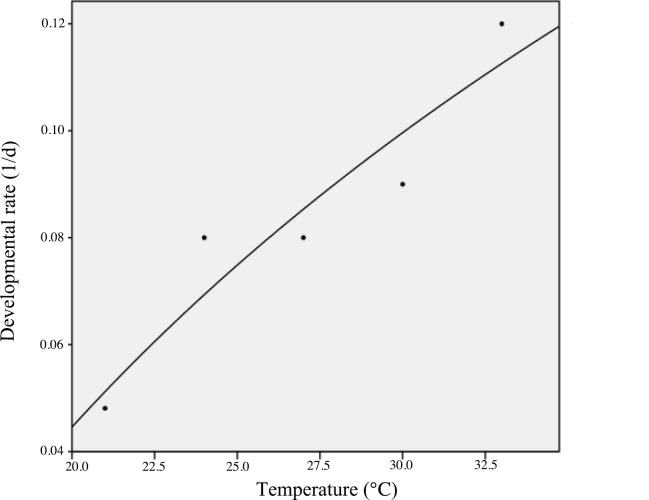
Developmental rate of the larval stage of *B*. *macroscopa* at different temperatures.

In the present study, the mean development rate of the larval stage fit the nonlinear equation: y = -0.362 + 0.136lg *x* with a coefficient of determination (*R*^2^) of 0.887 (Fig 1). Eggs in the 36°C treatment were unable to successfully hatch, therefore, the mean duration of the total pre-adult stages of *B*. *macroscopa* at different temperatures are only listed for temperatures between 21–33°C (Table 1). Significant differences (*P<0*.*05*) occurred in both the larval and total immature periods in the five different temperature treatments, with the shortest developmental time of the two periods being at 30°C and the longest at 21°C. The longest pupal period also occurred at 21°C while the shortest was in the 33°C treatment, although there was no significant difference (*P<0*.*05*) noted at the 30°C treatment. At 21°C, larvae required a seventh instar to complete their development, but needed only six instars in the24°C controls, and five when temperatures were between 27–33°C. Table 2 shows the adult longevity (from emergence to death of the males and females separately), the entire lifespan (from egg to adults’ death) and the mortality rates of *B*. *macroscopa* immature stages. The four different temperature settings (21, 24, 27, 30°C) used as rearing temperatures had a significant effect (*P<0*.*05*) on both the female and male longevities. However, neither the females nor the males had a statistical significance in longevity between the two temperature extremes, 33°C and 21°C. Each of the five temperature regimens had a significant impact (*P<0*.*05*) on the female lifespan, whereas there was no notable difference between male lifespans at 24°C and 27°C (*P>0*.*05*). The highest (31.43%) and lowest (15.33%) mortality rates occurred at 24°C and 27°C, respectively.

**Table 1:** The mean (±*SE*) duration of immature stages (days) of *B*. *macroscopa* reared at different temperatures under laboratory conditions.

	21°C	24°C	27°C	30°C	33°C
Incubation period	6.35±0.48a	5±0.00b	5±0.00b	3±0.00c	3±0.00c
1st instar period	1.36±0.05a	1.11±0.03b	1.85±0.04c	1.28±0.05a	1.10±0.02b
2nd instar period	2.48±0.06a	2.05±0.02b	1.96±0.03bc	2.80±0.04d	1.94±0.03c
3rd instar period	3.44±0.06a	3.17±0.04b	2.08±0.02c	1.92±0.02d	1.40±0.04e
4th instar period	2.83±0.08a	2.68±0.05a	2.20±0.05b	1.77±0.04c	2.67±0.03a
5th instar period	3.35±0.06a	3.58±0.15ab	4.41±0.08c	3.71±0.05b	2.98±0.06d
6th instar period	4.52±0.12	2.87±0.15	-	-	-
7th instar period	5.79±0.10	-	-	-	-
Larval period	20.79±0.26a	12.86±0.30b	12.47±0.20c	11.42±0.09d	8.47±0.11e
pupal period	8.15±0.10a	6.90±0.03b	5.04±0.03c	4.07±0.03d	4.06±0.02d
Total preadult	35.20±0.30a	24.65±0.32b	22.50±0.12c	18.57±0.10d	15.46±0.22e

Data in the table are marked as mean±*SE*. Means in the same row marked with different letters are significantly different at the 5% level using the LSD test.

**Table 2:** The means(±*SE*) of adult lifespan, longevity and mortality of immature stages (days) of *B*. *macroscopa* reared at different temperatures under laboratory conditions.

	21°C	24°C	27°C	30°C	33°C
Male adult longevity	26.62±0.92a	29.68±0.69b	34.17±1.46c	17.98±0.67d	25.29±1.05a
Female adult longevity	25.90±1.23a	26.14±0.96a	32.70±1.27b	17.41±0.65c	28.52±1.15a
Male entire lifespan	61.13±1.23a	56.55±0.77b	56.26±1.50b	36.73±0.70c	40.87±1.05d
Female entire lifespan	61.38±1.25a	49.26±1.00b	54.49±1.27c	35.80±0.64d	43.88±1.15e
Mortality of immature stages	24.66%	31.43%	15.33%	26.00%	27.52%

Data in the table are marked as mean±*SE*. Means in the same row marked with different letters are significantly different at the 5% level using the LSD test.

### Oviposition period and fecundity

Table 3 shows the APOP, TPOP, oviposition period and female fecundity values of *B*. *macroscopa* under different temperature conditions. Although the longest APOP value (3.81±0.29c) occurred at33°C the reverse was true in the TPOP values, where the shortest value (18.91±0.31c) occurred at 33°C. Females rearedat21°C had the highest fecundity (376.02±12.53a), whereas those from the 33°C treatment had the lowest (23.24±3.60c). The length of the oviposition period, in general, decreased from 21°C to 33°C.

**Table 3:** The mean (±*SE*) of ovipostion period (days) of *B*. *macroscopa* reared at different temperatures under laboratory conditions.

	21°C	24°C	27°C	30°C	33°C
APOP	2.55±0.15a	1.46±0.09b	1.45±0.12b	1.14±0.08b	3.81±0.29c
TPOP	37.88±0.41a	24.55±0.44b	24.23±0.18b	19.51±0.16c	18.91±0.31c
Oviposition period	19.06±0.86a	15.40±0.62b	18.31±0.51a	11.94±0.44c	11.22±0.55c
Total fecundity/female	376.02±12.53a	299.17±18.03b	299.18±11.43b	321.28±10.40b	23.24±3.60c

Data in the table are marked as mean±*SE*. Means in the same row marked with different letters are significantly different at the 5% level using the LSD test.

### Life table analysis

**Fig 2:**
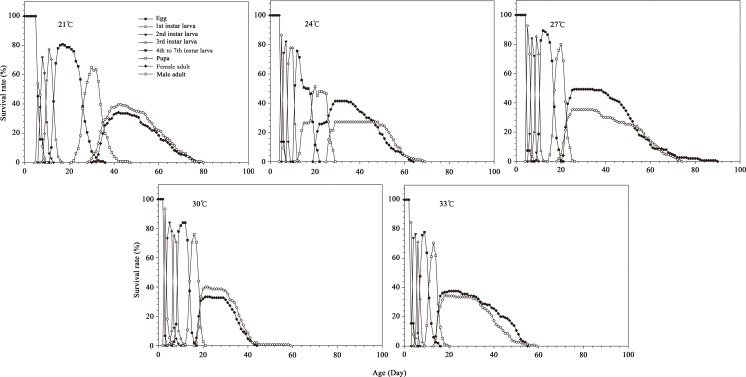
Age-stage-specific survival rates (*S*_*xj*_) of *B*. *macroscopa* reared at different temperatures under laboratory conditions.

**Fig 3:**
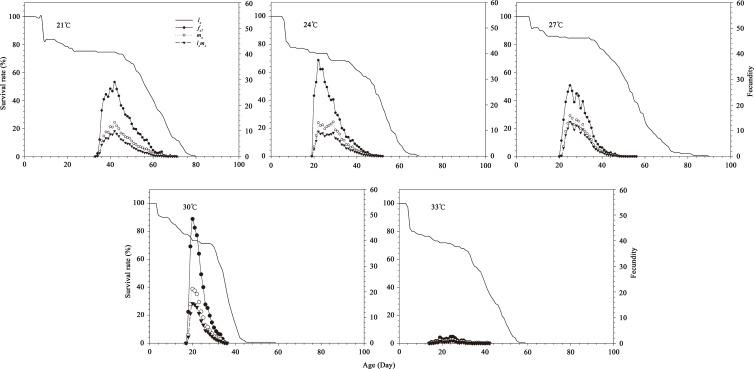
Age-specific survival rate (*l*_*x*_), age-specific fecundity (*m*_*x*_), age-specific mortality (*l*_*x*_*m*_*x*_) and age-stage-specific fecundity (*f*_*x*8_) of *B*. *macroscopa* reared at different temperatures under laboratory conditions.

The age-stage specific survival rate (*s*_*xj*_) values for insects cultured at various temperatures are shown in Fig 2. The lowest survival rate for the egg and larva stage was at 33°C, while the 27°C group had the highest survival rate for the egg and female pupal stage. The two remaining stages (larva and male pupa) exhibited the highest survival rate in the 30°C treatment. Fig 3 lists the age-specific survival rate, age-stage-specific fecundity (*f*_*x8*_) and age-specific fecundity (*m*_*x*_) values of *B*. *macroscopa* reared at the five different temperatures. Since only female adults (the tenth stage) produce offspring, there is only a single curve for the female age-stage-specific fecundity (*f*_*x8*_). The peak age stage-specific fecundities observed at the 21, 24, 27, 30, and 33°C temperatures occurred at 42, 29, 25, 20, and 25 days of age respectively, with corresponding values of 29.02, 37.44, 15.93, 21.12 and 2.49 eggs/female/day,. The age-specific fecundities in the five temperature treatments ranging from the highest to lowest fecundity values were found at24, 27, 30, 21, and 33°C. Table 4 shows the values for the age-stage, two-sex life table parameters. The highest values in net reproductive rate (*R*_*0*_) and finite rate of increase (*λ*) were found in the 27°C group, while the lowest R_0_, gross reproductive rate (*GRR*), intrinsic rate of increase (*r*_*m*_), and λ values for *B*. *macroscopa* occurred in individuals reared at 33°C. The longest and shortest mean generation times (*T*) for *B*. *macroscopa* occurred in the 21°C and 30°C treatments, respectively.

**Table 4:** The mean ±*SE* of life table parameters (days) of *B*. *macroscopa* reared in different temperatures under laboratory conditions.

	21°C	24°C	27°C	30°C	33°C
*R*_0_	121.05±15.16a	124.59±14.61a	147.60±13.46a	107.09a	8.43b
*GRR*	175.2±20.05a	178.23±18.50a	175.98±14.80a	148.16±16.25a	12.03±2.18b
*r*	0.11±0.003a	0.18±0.005b	0.17±0.003b	0.20±0.005c	0.08±0.008d
λ	1.12±0.003a	1.19±0.006b	1.19±0.004b	1.23±0.007c	1.09±0.009d
T	43.79±0.33a	27.3±0.41b	28.76±0.23c	22.96±0.16d	23.94±0.41e

Data in the table are marked as mean±*SE*. Means in the same row marked with different letters are significantly different at the 5% level using the LSD test.

## Discussion

A complete understanding of the population dynamics of the target species is essential to developing viable integrated control programs [23,24]. Life tables offer an effective means of tracking changes in a population’s growth as well as many other important parameters [25]. In population ecology studies involving insects, life tables offer the most integrated information on their growth, survival, reproduction and population growth parameters [26]. Many life table parameters including development, mortality, reproduction and other elements can be temperature-dependent [27–30]. Temperature is considered to be the most crucial abiotic factor affecting the establishment and growth of pest populations [31]. Temperature has proven to be a vital factor in an insect’s development and survival, with relatively minor variations having disproportional effects on the growth rate of one or more of their stages [32, 33]. Understanding the relationship between temperature variations and their effect on population dynamics would be invaluable in predicting the behavior of a pest on its host crop cultivars.

In order to represent temperature-dependent developmental rates, using linear or non-linear regression equations are effective means for demonstrating the relationship of development rate on temperature changes. In this study, we used 21, 24, 27, 30, 33 and 36°C to examine the effects that various temperatures have on the development of *B*. *macroscopa*. The 36°C temperature was apparently lethal to *B*. *macroscopa* -based on our observation that eggs never hatched at this temperature. Our results verified the sensitivity of this moth to high laboratory temperatures [34]. The female adults were capable of reproducing at all other temperatures (21–33°C), even when the 33°C group had an amazing low fecundity compared to the remaining four groups. Similarly, *Spodoptera exigua* Hübner was reported to experience suppression of growth and development at 32°C, whereas it could successfully complete its development from 20–29°C [35]. The developmental and metabolic rates apparently increase as the temperature is raised, however, as the temperature approaches the upper lethal limit, the metabolic rate decreases after the developmental rate [36]. The fecundity of *B*. *macroscopa* was a vital parameter that was directly related to variations in rearing temperatures. This phenomenon has been recorded in various other insect species, where increases in culturing temperatures often result in noticeable decreases in female productivity [37, 38].

There were distinct variations in the number of larval instars reared at diverse temperatures. The numbers of larval instars reached seven in the 21°C cohort, decreased to six at 24°C, and at higher temperatures (27–33°C) had only five instars. The discrepancies in the number of instars were likely related to the rearing conditions [22]. Since the immature stages are hypersensitive to abiotic and biotic elements in their environment, there is constant pressure on them to complete their immature stages as rapidly as possible to reach the adult stage and onset of maturity [21]. In our study, although the developmental time in the 21°C rearing environment was significantly longer than at other temperatures, the larvae had more opportunity to prey. Which in itself may increase their effectiveness as a biological control in the Integrated Pest Management (IPM) program [39, 40]. Nevertheless, the shorter developmental durations experienced at higher temperatures may sharply accelerate the immature stages, causing an early onset of maturity, which, in turn, would lead to increases in progeny and larger populations.

The small size of the eggs produced by *B*. *macroscopa*, making them difficult to observe in the field, coupled with the fact that the immature stages of this leaf folding pest develop entirely in the revolute leaves, makes direct observation of the moths’ development challenging. Establishing age-specific life tables based on statistics gained from laboratory studies would be of fundamental importance in creating integrated pest management programs for this important pest. In most insect populations, variations in developmental rates between individuals and among the sexes commonly occur. The stage distinction would not be detected if traditional age-specific female life tables had been used [41–43]. Realizing that the susceptibility of insects to varied environmental factors, pesticides, natural enemies and so on, often differs depending on their developmental stage, information regarding the population stage structure is critical to effective pest management [44]. This variation is not accounted for in traditional life table analysis and usually results in erroneous data conclusions [45]. Development of lab generated age-specific life tables can also play an important role in developing pest-resistant cultivars—one of the most environmentally friendly approaches of integrated pest management in reducing the negative effects produced by phytophagous insect pests [46].

In this research, the bootstrap technique was used to estimate the means and variance data of the population parameters. In our results, the highest *R*_*0*_ value for *B*. *macroscopa* (147.60) occurred at the27°C temperature group. Among the various population parameters, the intrinsic rate of increase (*r*_*m*_) is a critical demographic element for determining levels of environment resistance to insects [47]. Comparisons of the values within the *R*_*0*_ and *r*_*m*_ parameters invariably available for considerable insight beyond that from the independent analysis of individual life-history parameters [48]. Conversely, the decreased values obtained in the 33°C treatment revealed that this temperature was near the maximum developmental threshold for the pest. Consequently, *B*. *macroscopa* would be most likely to reach maximum population levels at 27°C.

The study establish baseline constant-temperature age-stage two-sex life tables for the developmental and survival parameters of the *B*. *macroscopa*, which could be available to model development in the wild and estimate potential distribution limits. Knowledge of the temperature-dependent growth and development of *B*. *macroscopa* could be used to forecast the peak periods as well as reduced activity of this pest, which could inform control programs. In addition. key bioclimatic parameters such as the reproductive growth under different temperatures could be used to optimize the production methods in mass rearing for parasitoids.

Further studies are needed to identify the phytochemicals responsible for retarding development at the resistant temperatures in order to design a more effective IPM program for *B*. *macroscopa* in the field.

## Supporting information

S1 Data Set Developmental rate of the larval stage of *B*. *macroscopa* at different temperatures. (DOC)

S2 Data Set Age-stage-specific survival rates (*S*_*xj*_) of *B*. *macroscopa* reared at different temperatures under laboratory conditions. (DOC)

S3 Data Set Age-specific survival rate (*l*_*x*_), age-specific fecundity (*m*_*x*_), age-specific mortality (*l*_*x*_*m*_*x*_) and age-stage-specific fecundity (*f*_*x8*_) of *B*. *macroscopa* reared at different temperatures under laboratory conditions. (DOC)
