# Supplementary material for: Temperature effects on development and fecundity of Brachmia macroscopa (Lepidoptera: Gelechiidae)
Source: PLoS One. 2017 Mar 2;12(3):e0173065. doi: 10.1371/journal.pone.0173065 (PMC5333877; doi:10.1371/journal.pone.0173065)
Supplement: S1 Data Set — (DOC) [file pone.0173065.s001.doc]

**Data Set Fig. 1. Developmental rate of the larval stage of *B. macroscopa* at different temperatures**

| Temperature | Larval duration (d) | Developmtal rate (1/d) |
| --- | --- | --- |
| 21°C | 20.79 | 0.0481 |
| 24°C | 12.86 | 0.0778 |
| 27°C | 12.47 | 0.0802 |
| 30°C | 11.42 | 0.0876 |
| 33°C | 8.47 | 0.1181 |
