# Supplementary material for: Temperature effects on development and fecundity of Brachmia macroscopa (Lepidoptera: Gelechiidae)
Source: PLoS One. 2017 Mar 2;12(3):e0173065. doi: 10.1371/journal.pone.0173065 (PMC5333877; doi:10.1371/journal.pone.0173065)
Supplement: S2 Data Set — (DOC) [file pone.0173065.s002.doc]

**Data Set Fig. 2. Age-stage-specific survival rates (*S_xj_*) of *B. macroscopa* reared at different temperatures under laboratory conditions**

**21℃**

| 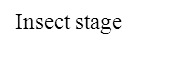   | 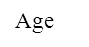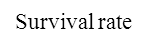 | | --- | | | Egg | L1 | L2 | L3 | L4-7 | Pupa | Female | Male |
| --- | --- | --- | --- | --- | --- | --- | --- | --- | --- |
|
| 0 | 1 |  |  |  |  |  |  |  |
| 1 | 1 |  |  |  |  |  |  |  |
| 2 | 1 |  |  |  |  |  |  |  |
| 3 | 1 |  |  |  |  |  |  |  |
| 4 | 1 |  |  |  |  |  |  |  |
| 5 | 1 | 0 |  |  |  |  |  |  |
| 6 | 0.4521 | 0.5411 | 0 |  |  |  |  |  |
| 7 | 0.1575 | 0.4589 | 0.3767 | 0 |  |  |  |  |
| 8 | 0.1575 | 0.1096 | 0.7192 | 6.85E-03 |  |  |  |  |
| 9 | 0 | 0.0274 | 0.6096 | 0.1986 |  |  |  |  |
| 10 |  | 0 | 0.274 | 0.5616 |  |  |  |  |
| 11 |  |  | 0.0616 | 0.774 | 0 |  |  |  |
| 12 |  |  | 0.0274 | 0.7055 | 0.1027 |  |  |  |
| 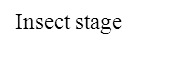   | 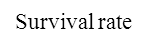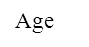 | | --- | | | Egg | L1 | L2 | L3 | L4-7 | Pupa | Female | Male |
|
| 13 |  |  | 0 | 0.4178 | 0.4178 |  |  |  |
| 14 |  |  |  | 0.1575 | 0.6781 |  |  |  |
| 15 |  |  |  | 0.0411 | 0.7808 |  |  |  |
| 16 |  |  |  | 0.0137 | 0.8014 |  |  |  |
| 17 |  |  |  | 0 | 0.8082 |  |  |  |
| 18 |  |  |  |  | 0.8014 |  |  |  |
| 19 |  |  |  |  | 0.7877 |  |  |  |
| 20 |  |  |  |  | 0.7877 |  |  |  |
| 21 |  |  |  |  | 0.774 | 0 |  |  |
| 22 |  |  |  |  | 0.7603 | 0.0137 |  |  |
| 23 |  |  |  |  | 0.6918 | 0.0616 |  |  |
| 24 |  |  |  |  | 0.6575 | 0.0959 |  |  |
| 25 |  |  |  |  | 0.5685 | 0.1849 |  |  |
| 26 |  |  |  |  | 0.3836 | 0.3699 |  |  |
| 27 |  |  |  |  | 0.2808 | 0.4726 |  |  |
| 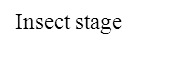   | 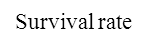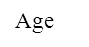 | | --- | | | Egg | L1 | L2 | L3 | L4-7 | Pupa | Female | Male |
|
| 28 |  |  |  |  | 0.2055 | 0.5479 |  | 0 |
|  |  |  |  |  |  |  |  |  |
| 29 |  |  |  |  | 0.1438 | 0.6027 |  | 6.85E-03 |
| 30 |  |  |  |  | 0.0753 | 0.6507 | 0 | 0.0274 |
| 31 |  |  |  |  | 0.0548 | 0.6301 | 6.85E-03 | 0.0616 |
| 32 |  |  |  |  | 0.0342 | 0.637 | 6.85E-03 | 0.0753 |
| 33 |  |  |  |  | 0.0137 | 0.5548 | 0.0548 | 0.1301 |
| 34 |  |  |  |  | 6.85E-03 | 0.3973 | 0.1301 | 0.2123 |
| 35 |  |  |  |  | 6.85E-03 | 0.274 | 0.1986 | 0.2671 |
| 36 |  |  |  |  | 0 | 0.1986 | 0.2534 | 0.2945 |
| 37 |  |  |  |  |  | 0.1712 | 0.2671 | 0.3082 |
| 38 |  |  |  |  |  | 0.1027 | 0.2945 | 0.3493 |
| 39 |  |  |  |  |  | 0.0685 | 0.3014 | 0.3767 |
| 40 |  |  |  |  |  | 0.0411 | 0.3288 | 0.3767 |
| 41 |  |  |  |  |  | 0.0274 | 0.3356 | 0.3836 |
| 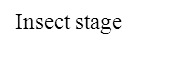   | 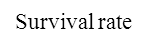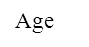 | | --- | | | Egg | L1 | L2 | L3 | L4-7 | Pupa | Female | Male |
|
| 42 |  |  |  |  |  | 6.85E-03 | 0.3425 | 0.3973 |
| 43 |  |  |  |  |  | 6.85E-03 | 0.3356 | 0.3973 |
| 44 |  |  |  |  |  | 6.85E-03 | 0.3356 | 0.3904 |
| 45 |  |  |  |  |  | 6.85E-03 | 0.3356 | 0.3904 |
| 46 |  |  |  |  |  | 6.85E-03 | 0.3356 | 0.3836 |
| 47 |  |  |  |  |  | 0 | 0.3288 | 0.3699 |
| 48 |  |  |  |  |  |  | 0.3219 | 0.363 |
| 49 |  |  |  |  |  |  | 0.3219 | 0.3562 |
| 50 |  |  |  |  |  |  | 0.3151 | 0.3493 |
| 51 |  |  |  |  |  |  | 0.3082 | 0.3493 |
| 52 |  |  |  |  |  |  | 0.274 | 0.3425 |
| 53 |  |  |  |  |  |  | 0.2603 | 0.3425 |
| 54 |  |  |  |  |  |  | 0.2534 | 0.3288 |
| 55 |  |  |  |  |  |  | 0.2466 | 0.3014 |
| 56 |  |  |  |  |  |  | 0.2329 | 0.2808 |
| 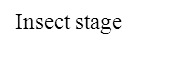   | 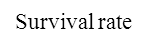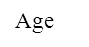 | | --- | | | Egg | L1 | L2 | L3 | L4-7 | Pupa | Female | Male |
|
| 57 |  |  |  |  |  |  | 0.2192 | 0.2671 |
| 58 |  |  |  |  |  |  | 0.2192 | 0.2397 |
| 59 |  |  |  |  |  |  | 0.2055 | 0.226 |
| 60 |  |  |  |  |  |  | 0.1849 | 0.2192 |
| 61 |  |  |  |  |  |  | 0.1575 | 0.2123 |
| 62 |  |  |  |  |  |  | 0.1507 | 0.1849 |
| 63 |  |  |  |  |  |  | 0.1507 | 0.1712 |
| 64 |  |  |  |  |  |  | 0.137 | 0.1644 |
| 65 |  |  |  |  |  |  | 0.1233 | 0.1507 |
| 66 |  |  |  |  |  |  | 0.1027 | 0.1301 |
| 67 |  |  |  |  |  |  | 0.0822 | 0.1164 |
| 68 |  |  |  |  |  |  | 0.0753 | 0.089 |
| 69 |  |  |  |  |  |  | 0.0616 | 0.0753 |
| 70 |  |  |  |  |  |  | 0.0548 | 0.0753 |
| 71 |  |  |  |  |  |  | 0.0548 | 0.0616 |
| 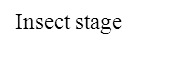   | 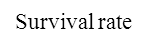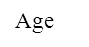 | | --- | | | Egg | L1 | L2 | L3 | L4-7 | Pupa | Female | Male |
|
| 72 |  |  |  |  |  |  | 0.0479 | 0.0616 |
| 73 |  |  |  |  |  |  | 0.0411 | 0.0411 |
| 74 |  |  |  |  |  |  | 0.0342 | 0.0274 |
| 75 |  |  |  |  |  |  | 0.0205 | 0.0137 |
| 76 |  |  |  |  |  |  | 0.0137 | 6.85E-03 |
| 77 |  |  |  |  |  |  | 6.85E-03 | 6.85E-03 |
| 78 |  |  |  |  |  |  | 0 | 6.85E-03 |
| 79 |  |  |  |  |  |  |  | 6.85E-03 |
| 80 |  |  |  |  |  |  |  | 0 |

**24**℃

| 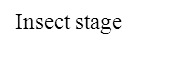   | 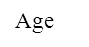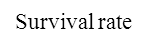 | | --- | | | Egg | L1 | L2 | L3 | L4-7 | Pupa | Female | Male |
| --- | --- | --- | --- | --- | --- | --- | --- | --- | --- |
|
| 0 | 1 |  |  |  |  |  |  |  |
| 1 | 1 |  |  |  |  |  |  |  |
| 2 | 1 |  |  |  |  |  |  |  |
| 3 | 1 |  |  |  |  |  |  |  |
| 4 | 1 | 0 |  |  |  |  |  |  |
| 5 | 0.1357 | 0.8643 | 0 |  |  |  |  |  |
| 6 | 0.1357 | 0.0929 | 0.7429 |  |  |  |  |  |
| 7 | 0 | 0 | 0.8214 | 0 |  |  |  |  |
| 8 |  |  | 0.1357 | 0.6714 |  |  |  |  |
| 9 |  |  | 0 | 0.7786 |  |  |  |  |
| 10 |  |  |  | 0.7786 | 0 |  |  |  |
| 11 |  |  |  | 0.2571 | 0.5214 | 0 |  |  |
| 12 |  |  |  | 7.14E-03 | 0.7571 | 7.14E-03 |  |  |
| 13 |  |  |  | 0 | 0.7143 | 0.0571 |  |  |
| 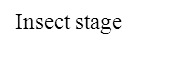   | 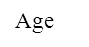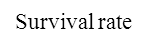 | | --- | | | Egg | L1 | L2 | L3 | L4-7 | Pupa | Female | Male |
|
| 14 |  |  |  |  | 0.5571 | 0.2143 |  |  |
| 15 |  |  |  |  | 0.5071 | 0.2643 |  |  |
| 16 |  |  |  |  | 0.5 | 0.2643 |  |  |
| 17 |  |  |  |  | 0.5 | 0.2643 |  |  |
| 18 |  |  |  |  | 0.4643 | 0.2786 |  |  |
| 19 |  |  |  |  | 0.2714 | 0.4714 | 0 |  |
| 20 |  |  |  |  | 0.1143 | 0.5143 | 0.1143 |  |
| 21 |  |  |  |  | 0.0786 | 0.45 | 0.2071 |  |
| 22 |  |  |  |  | 0 | 0.4786 | 0.2571 |  |
| 23 |  |  |  |  |  | 0.4786 | 0.2571 |  |
| 24 |  |  |  |  |  | 0.4714 | 0.2643 |  |
| 25 |  |  |  |  |  | 0.4643 | 0.2714 | 0 |
| 26 |  |  |  |  |  | 0.2714 | 0.3143 | 0.15 |
| 27 |  |  |  |  |  | 0.1286 | 0.3571 | 0.2143 |
| 28 |  |  |  |  |  | 0.0786 | 0.3929 | 0.2143 |
| 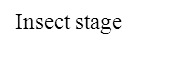   | 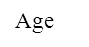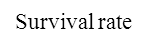 | | --- | | | Egg | L1 | L2 | L3 | L4-7 | Pupa | Female | Male |
|
| 29 |  |  |  |  |  | 0 | 0.4143 | 0.2714 |
| 30 |  |  |  |  |  |  | 0.4143 | 0.2714 |
| 31 |  |  |  |  |  |  | 0.4143 | 0.2714 |
| 32 |  |  |  |  |  |  | 0.4143 | 0.2714 |
| 33 |  |  |  |  |  |  | 0.4143 | 0.2714 |
| 34 |  |  |  |  |  |  | 0.4143 | 0.2714 |
| 35 |  |  |  |  |  |  | 0.4071 | 0.2714 |
| 36 |  |  |  |  |  |  | 0.4071 | 0.2714 |
| 37 |  |  |  |  |  |  | 0.3929 | 0.2714 |
| 38 |  |  |  |  |  |  | 0.3786 | 0.2714 |
| 39 |  |  |  |  |  |  | 0.3571 | 0.2714 |
| 40 |  |  |  |  |  |  | 0.3429 | 0.2714 |
| 41 |  |  |  |  |  |  | 0.3357 | 0.2714 |
| 42 |  |  |  |  |  |  | 0.3214 | 0.2714 |
| 43 |  |  |  |  |  |  | 0.3 | 0.2714 |
| 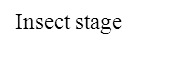   | 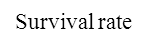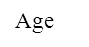 | | --- | | | Egg | L1 | L2 | L3 | L4-7 | Pupa | Female | Male |
|
| 44 |  |  |  |  |  |  | 0.3 | 0.2714 |
| 45 |  |  |  |  |  |  | 0.2857 | 0.2714 |
| 46 |  |  |  |  |  |  | 0.2786 | 0.2714 |
| 47 |  |  |  |  |  |  | 0.25 | 0.2714 |
| 48 |  |  |  |  |  |  | 0.2357 | 0.2714 |
| 49 |  |  |  |  |  |  | 0.1929 | 0.25 |
| 50 |  |  |  |  |  |  | 0.1857 | 0.25 |
| 51 |  |  |  |  |  |  | 0.1571 | 0.2429 |
| 52 |  |  |  |  |  |  | 0.1286 | 0.2214 |
| 53 |  |  |  |  |  |  | 0.1143 | 0.2 |
| 54 |  |  |  |  |  |  | 0.0929 | 0.1714 |
| 55 |  |  |  |  |  |  | 0.0857 | 0.15 |
| 56 |  |  |  |  |  |  | 0.0714 | 0.1143 |
| 57 |  |  |  |  |  |  | 0.0714 | 0.1 |
| 58 |  |  |  |  |  |  | 0.0643 | 0.0929 |
| 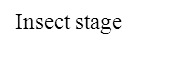   | 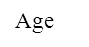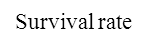 | | --- | | | Egg | L1 | L2 | L3 | L4-7 | Pupa | Female | Male |
|
| 59 |  |  |  |  |  |  | 0.05 | 0.0571 |
| 60 |  |  |  |  |  |  | 0.0429 | 0.0429 |
| 61 |  |  |  |  |  |  | 0.0357 | 0.0357 |
| 62 |  |  |  |  |  |  | 0.0143 | 0.0286 |
| 63 |  |  |  |  |  |  | 7.14E-03 | 0.0214 |
| 64 |  |  |  |  |  |  | 0 | 0.0214 |
| 65 |  |  |  |  |  |  |  | 0.0143 |
| 66 |  |  |  |  |  |  |  | 0.0143 |
| 67 |  |  |  |  |  |  |  | 0.0143 |
| 68 |  |  |  |  |  |  |  | 7.14E-03 |
| 69 |  |  |  |  |  |  |  | 0 |

**27℃**

| 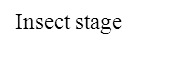   | 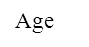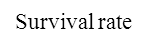 | | --- | | | Egg | L1 | L2 | L3 | L4-7 | Pupa | Female | Male |
| --- | --- | --- | --- | --- | --- | --- | --- | --- | --- |
|
| 0 | 1 |  |  |  |  |  |  |  |
| 1 | 1 |  |  |  |  |  |  |  |
| 2 | 1 |  |  |  |  |  |  |  |
| 3 | 1 |  |  |  |  |  |  |  |
| 4 | 1 | 0 |  |  |  |  |  |  |
| 5 | 0.0733 | 0.9267 | 0 |  |  |  |  |  |
| 6 | 0.0733 | 0.7333 | 0.1867 | 0 |  |  |  |  |
| 7 | 0 | 0.0467 | 0.84 | 0.0333 |  |  |  |  |
| 8 |  | 0 | 0.72 | 0.2 | 0 |  |  |  |
| 9 |  |  | 0.06 | 0.8533 | 6.67E-03 |  |  |  |
| 10 |  |  | 0 | 0.7333 | 0.1867 |  |  |  |
| 11 |  |  |  | 0.0733 | 0.8267 |  |  |  |
| 12 |  |  |  | 6.67E-03 | 0.8933 |  |  |  |
| 13 |  |  |  | 0 | 0.8867 |  |  |  |
| 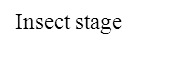   | 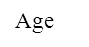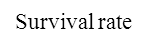 | | --- | | | Egg | L1 | L2 | L3 | L4-7 | Pupa | Female | Male |
|
| 14 |  |  |  |  | 0.8667 | 0 |  |  |
| 15 |  |  |  |  | 0.8133 | 0.0467 |  |  |
| 16 |  |  |  |  | 0.6867 | 0.1733 |  |  |
| 17 |  |  |  |  | 0.3733 | 0.4867 |  |  |
| 18 |  |  |  |  | 0.16 | 0.7 |  | 0 |
| 19 |  |  |  |  | 0.08 | 0.76 |  | 0.02 |
| 20 |  |  |  |  | 6.67E-03 | 0.8 | 0 | 0.0467 |
| 21 |  |  |  |  | 0 | 0.6867 | 0.04 | 0.1267 |
| 22 |  |  |  |  |  | 0.4067 | 0.2333 | 0.2133 |
| 23 |  |  |  |  |  | 0.16 | 0.3933 | 0.3 |
| 24 |  |  |  |  |  | 0.0933 | 0.4333 | 0.32 |
| 25 |  |  |  |  |  | 6.67E-03 | 0.4867 | 0.3533 |
| 26 |  |  |  |  |  | 0 | 0.4933 | 0.3533 |
| 27 |  |  |  |  |  |  | 0.4933 | 0.3533 |
| 28 |  |  |  |  |  |  | 0.4933 | 0.3533 |
| 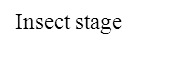   | 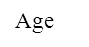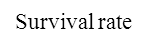 | | --- | | | Egg | L1 | L2 | L3 | L4-7 | Pupa | Female | Male |
|
| 29 |  |  |  |  |  |  | 0.4933 | 0.3533 |
| 30 |  |  |  |  |  |  | 0.4933 | 0.3533 |
| 31 |  |  |  |  |  |  | 0.4933 | 0.3533 |
| 32 |  |  |  |  |  |  | 0.4933 | 0.3533 |
| 33 |  |  |  |  |  |  | 0.4933 | 0.3533 |
| 34 |  |  |  |  |  |  | 0.4933 | 0.3533 |
| 35 |  |  |  |  |  |  | 0.4867 | 0.3467 |
| 36 |  |  |  |  |  |  | 0.4867 | 0.3467 |
| 37 |  |  |  |  |  |  | 0.4867 | 0.3333 |
| 38 |  |  |  |  |  |  | 0.4867 | 0.3133 |
| 39 |  |  |  |  |  |  | 0.4733 | 0.3 |
| 40 |  |  |  |  |  |  | 0.4667 | 0.3 |
| 41 |  |  |  |  |  |  | 0.4533 | 0.3 |
| 42 |  |  |  |  |  |  | 0.4333 | 0.3 |
| 43 |  |  |  |  |  |  | 0.4267 | 0.2933 |
| 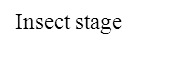   | 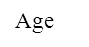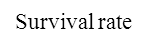 | | --- | | | Egg | L1 | L2 | L3 | L4-7 | Pupa | Female | Male |
|
| 44 |  |  |  |  |  |  | 0.42 | 0.28 |
| 45 |  |  |  |  |  |  | 0.4133 | 0.2733 |
| 46 |  |  |  |  |  |  | 0.4067 | 0.2667 |
| 47 |  |  |  |  |  |  | 0.3867 | 0.2667 |
| 48 |  |  |  |  |  |  | 0.3667 | 0.2667 |
| 49 |  |  |  |  |  |  | 0.3467 | 0.2533 |
| 50 |  |  |  |  |  |  | 0.32 | 0.2533 |
| 51 |  |  |  |  |  |  | 0.2933 | 0.2533 |
| 52 |  |  |  |  |  |  | 0.2733 | 0.2467 |
| 53 |  |  |  |  |  |  | 0.2267 | 0.24 |
| 54 |  |  |  |  |  |  | 0.2133 | 0.24 |
| 55 |  |  |  |  |  |  | 0.18 | 0.22 |
| 56 |  |  |  |  |  |  | 0.1733 | 0.2133 |
| 57 |  |  |  |  |  |  | 0.1667 | 0.1933 |
| 58 |  |  |  |  |  |  | 0.1533 | 0.16 |
| 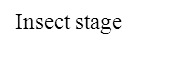   | 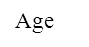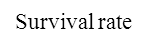 | | --- | | | Egg | L1 | L2 | L3 | L4-7 | Pupa | Female | Male |
|
| 59 |  |  |  |  |  |  | 0.1467 | 0.16 |
| 60 |  |  |  |  |  |  | 0.1133 | 0.14 |
| 61 |  |  |  |  |  |  | 0.0933 | 0.1267 |
| 62 |  |  |  |  |  |  | 0.0867 | 0.1133 |
| 63 |  |  |  |  |  |  | 0.0867 | 0.1 |
| 64 |  |  |  |  |  |  | 0.0867 | 0.08 |
| 65 |  |  |  |  |  |  | 0.0867 | 0.0667 |
| 66 |  |  |  |  |  |  | 0.08 | 0.0533 |
| 67 |  |  |  |  |  |  | 0.08 | 0.0467 |
| 68 |  |  |  |  |  |  | 0.06 | 0.0467 |
| 69 |  |  |  |  |  |  | 0.0533 | 0.04 |

**30℃**

| 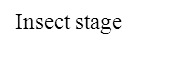   | 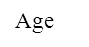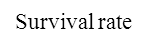 | | --- | | | Egg | L1 | L2 | L3 | L4-7 | Pupa | Female | Male |
| --- | --- | --- | --- | --- | --- | --- | --- | --- | --- |
|
| 0 | 1 |  |  |  |  |  |  |  |
| 1 | 1 |  |  |  |  |  |  |  |
| 2 | 1 | 0 |  |  |  |  |  |  |
| 3 | 0.0667 | 0.9333 | 0 |  |  |  |  |  |
| 4 | 0 | 0.18 | 0.7333 | 0 |  |  |  |  |
| 5 |  | 0.0533 | 0.84 | 0.0133 |  |  |  |  |
| 6 |  | 0.02 | 0.78 | 0.1 | 0 |  |  |  |
| 7 |  | 0 | 0.12 | 0.7533 | 0.0267 |  |  |  |
| 8 |  |  | 0.0467 | 0.7067 | 0.1467 |  |  |  |
| 9 |  |  | 0.0133 | 0.1 | 0.78 |  |  |  |
| 10 |  |  | 0 | 0.0467 | 0.82 |  |  |  |
| 11 |  |  |  | 0.0133 | 0.84 | 0 |  |  |
| 12 |  |  |  | 0 | 0.84 | 6.67E-03 |  |  |
| 13 |  |  |  |  | 0.72 | 0.1067 |  |  |
| 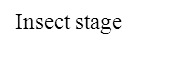   | 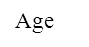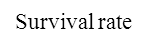 | | --- | | | Egg | L1 | L2 | L3 | L4-7 | Pupa | Female | Male |
|
| 14 |  |  |  |  | 0.3733 | 0.44 |  |  |
| 15 |  |  |  |  | 0.0867 | 0.7067 |  |  |
| 16 |  |  |  |  | 0.02 | 0.76 | 0 | 0 |
| 17 |  |  |  |  | 0 | 0.7067 | 0.0533 | 0.02 |
| 18 |  |  |  |  |  | 0.44 | 0.2 | 0.1333 |
| 19 |  |  |  |  |  | 0.12 | 0.3067 | 0.3333 |
| 20 |  |  |  |  |  | 0.0267 | 0.32 | 0.3867 |
| 21 |  |  |  |  |  | 0 | 0.3333 | 0.4 |
| 22 |  |  |  |  |  |  | 0.3333 | 0.4 |
| 23 |  |  |  |  |  |  | 0.3333 | 0.3933 |
| 24 |  |  |  |  |  |  | 0.3267 | 0.3867 |
| 25 |  |  |  |  |  |  | 0.3267 | 0.3867 |
| 26 |  |  |  |  |  |  | 0.3267 | 0.3867 |
| 27 |  |  |  |  |  |  | 0.3267 | 0.3867 |
| 28 |  |  |  |  |  |  | 0.3267 | 0.3867 |
| 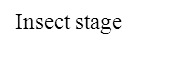   | 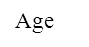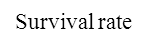 | | --- | | | Egg | L1 | L2 | L3 | L4-7 | Pupa | Female | Male |
|
| 29 |  |  |  |  |  |  | 0.3267 | 0.38 |
| 30 |  |  |  |  |  |  | 0.3133 | 0.3733 |
| 31 |  |  |  |  |  |  | 0.2933 | 0.3533 |
| 32 |  |  |  |  |  |  | 0.2733 | 0.3133 |
| 33 |  |  |  |  |  |  | 0.2467 | 0.3067 |
| 34 |  |  |  |  |  |  | 0.2267 | 0.2933 |
| 35 |  |  |  |  |  |  | 0.18 | 0.26 |
| 36 |  |  |  |  |  |  | 0.1533 | 0.2067 |
| 37 |  |  |  |  |  |  | 0.1133 | 0.1733 |
| 38 |  |  |  |  |  |  | 0.0933 | 0.14 |
| 39 |  |  |  |  |  |  | 0.08 | 0.1 |
| 40 |  |  |  |  |  |  | 0.0467 | 0.0867 |
| 41 |  |  |  |  |  |  | 0.0333 | 0.0533 |
| 42 |  |  |  |  |  |  | 0.02 | 0.02 |
| 43 |  |  |  |  |  |  | 6.67E-03 | 0.02 |
| 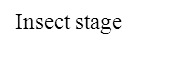   | 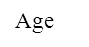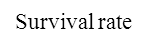 | | --- | | | Egg | L1 | L2 | L3 | L4-7 | Pupa | Female | Male |
|
| 44 |  |  |  |  |  |  | 0 | 0.02 |
| 45 |  |  |  |  |  |  |  | 6.67E-03 |
| 46 |  |  |  |  |  |  |  | 6.67E-03 |
| 47 |  |  |  |  |  |  |  | 6.67E-03 |
| 48 |  |  |  |  |  |  |  | 6.67E-03 |
| 49 |  |  |  |  |  |  |  | 6.67E-03 |
| 50 |  |  |  |  |  |  |  | 6.67E-03 |
| 51 |  |  |  |  |  |  |  | 6.67E-03 |
| 52 |  |  |  |  |  |  |  | 6.67E-03 |
| 53 |  |  |  |  |  |  |  | 6.67E-03 |
| 54 |  |  |  |  |  |  |  | 6.67E-03 |
| 55 |  |  |  |  |  |  |  | 6.67E-03 |
| 56 |  |  |  |  |  |  |  | 6.67E-03 |
| 57 |  |  |  |  |  |  |  | 6.67E-03 |
| 58 |  |  |  |  |  |  |  | 6.67E-03 |
| 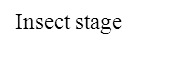   | 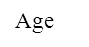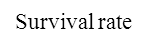 | | --- | | | Egg | L1 | L2 | L3 | L4-7 | Pupa | Female | Male |
|
| 59 |  |  |  |  |  |  |  | 0 |

**33℃**

| 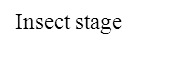   | 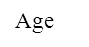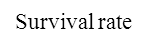 | | --- | | | Egg | L1 | L2 | L3 | L4-7 | Pupa | Female | Male |
| --- | --- | --- | --- | --- | --- | --- | --- | --- | --- |
|
| 0 | 1 |  |  |  |  |  |  |  |
| 1 | 1 |  |  |  |  |  |  |  |
| 2 | 1 |  |  |  |  |  |  |  |
| 3 | 0.1544 | 0.8456 | 0 |  |  |  |  |  |
| 4 | 0.1544 | 0.0805 | 0.7383 | 0 |  |  |  |  |
| 5 | 0 | 0 | 0.7651 | 0.0537 |  |  |  |  |
| 6 |  |  | 0.0872 | 0.7114 | 0 |  |  |  |
| 7 |  |  | 0 | 0.3221 | 0.4765 |  |  |  |
| 8 |  |  |  | 0.0268 | 0.7584 |  |  |  |
| 9 |  |  |  | 0 | 0.7785 | 0 |  |  |
| 10 |  |  |  |  | 0.6376 | 0.1409 |  |  |
| 11 |  |  |  |  | 0.3289 | 0.443 |  |  |
| 12 |  |  |  |  | 0.1544 | 0.6107 |  |  |
| 13 |  |  |  |  | 0.0604 | 0.7047 | 0 | 0 |
| 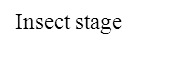   | 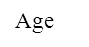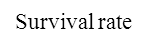 | | --- | | | Egg | L1 | L2 | L3 | L4-7 | Pupa | Female | Male |
|
| 14 |  |  |  |  | 0.0268 | 0.6174 | 0.0671 | 0.0537 |
| 15 |  |  |  |  | 0.0201 | 0.2886 | 0.2349 | 0.2081 |
| 16 |  |  |  |  | 0 | 0.1275 | 0.3423 | 0.2685 |
| 17 |  |  |  |  |  | 0.0537 | 0.3624 | 0.3154 |
| 18 |  |  |  |  |  | 0.0134 | 0.3691 | 0.349 |
| 19 |  |  |  |  |  | 6.71E-03 | 0.3691 | 0.3423 |
| 20 |  |  |  |  |  | 0 | 0.3758 | 0.3423 |
| 21 |  |  |  |  |  |  | 0.3758 | 0.3423 |
| 22 |  |  |  |  |  |  | 0.3758 | 0.3423 |
| 23 |  |  |  |  |  |  | 0.3758 | 0.3356 |
| 24 |  |  |  |  |  |  | 0.3758 | 0.3356 |
| 25 |  |  |  |  |  |  | 0.3691 | 0.3356 |
| 26 |  |  |  |  |  |  | 0.3557 | 0.3356 |
| 27 |  |  |  |  |  |  | 0.3557 | 0.3356 |
| 28 |  |  |  |  |  |  | 0.3557 | 0.3356 |
| 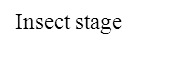   | 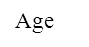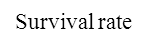 | | --- | | | Egg | L1 | L2 | L3 | L4-7 | Pupa | Female | Male |
|
| 29 |  |  |  |  |  |  | 0.3423 | 0.3356 |
| 30 |  |  |  |  |  |  | 0.3423 | 0.3356 |
| 31 |  |  |  |  |  |  | 0.3423 | 0.3221 |
| 32 |  |  |  |  |  |  | 0.3356 | 0.3221 |
| 33 |  |  |  |  |  |  | 0.3221 | 0.3087 |
| 34 |  |  |  |  |  |  | 0.302 | 0.2819 |
| 35 |  |  |  |  |  |  | 0.2886 | 0.2617 |
| 36 |  |  |  |  |  |  | 0.2886 | 0.255 |
| 37 |  |  |  |  |  |  | 0.2819 | 0.2416 |
| 38 |  |  |  |  |  |  | 0.2819 | 0.2282 |
| 39 |  |  |  |  |  |  | 0.2617 | 0.2081 |
| 40 |  |  |  |  |  |  | 0.255 | 0.1745 |
| 41 |  |  |  |  |  |  | 0.2416 | 0.1477 |
| 42 |  |  |  |  |  |  | 0.2215 | 0.1342 |
| 43 |  |  |  |  |  |  | 0.2013 | 0.1275 |
| 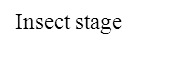   | 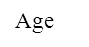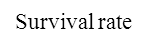 | | --- | | | Egg | L1 | L2 | L3 | L4-7 | Pupa | Female | Male |
|
| 44 |  |  |  |  |  |  | 0.2013 | 0.1074 |
| 45 |  |  |  |  |  |  | 0.1946 | 0.0872 |
| 46 |  |  |  |  |  |  | 0.1879 | 0.0872 |
| 47 |  |  |  |  |  |  | 0.1745 | 0.0671 |
| 48 |  |  |  |  |  |  |  | 6.67E-03 |
| 49 |  |  |  |  |  |  |  | 6.67E-03 |
| 50 |  |  |  |  |  |  |  | 6.67E-03 |
| 51 |  |  |  |  |  |  |  | 6.67E-03 |
| 52 |  |  |  |  |  |  |  | 6.67E-03 |
| 53 |  |  |  |  |  |  |  | 6.67E-03 |
| 54 |  |  |  |  |  |  |  | 6.67E-03 |
| 55 |  |  |  |  |  |  |  | 6.67E-03 |
| 56 |  |  |  |  |  |  |  | 6.67E-03 |
| 57 |  |  |  |  |  |  |  | 6.67E-03 |
| 58 |  |  |  |  |  |  |  | 6.67E-03 |
| 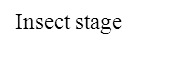   | 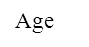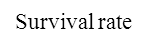 | | --- | | | Egg | L1 | L2 | L3 | L4-7 | Pupa | Female | Male |
|
| 59 |  |  |  |  |  |  |  | 0 |
