# Supplementary material for: Temperature effects on development and fecundity of Brachmia macroscopa (Lepidoptera: Gelechiidae)
Source: PLoS One. 2017 Mar 2;12(3):e0173065. doi: 10.1371/journal.pone.0173065 (PMC5333877; doi:10.1371/journal.pone.0173065)
Supplement: S3 Data Set — (DOC) [file pone.0173065.s003.doc]

**Data Set Fig. 3. Age-specific survival rate (*l_x_*), age-specific fecundity (*m_x_*), age-specific mortality (*l_x_m_x_*) and age-stage-specific fecundity (*f_x7_*) of *B. macroscopa* reared at different temperatures under laboratory conditions**


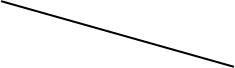
**21℃**

| 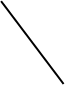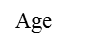   | 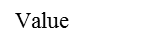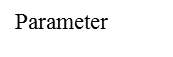 | | --- | | | *l_x_* | *f_x7_* | *m_x_* | *l_x_m_x_* |
| --- | --- | --- | --- | --- | --- |
|
| 0 | 1.0000 |  |  |  |
| 1 | 1.0000 |  |  |  |
| 2 | 1.0000 |  |  |  |
| 3 | 1.0000 |  |  |  |
| 4 | 1.0000 |  |  |  |
| 5 | 1.0000 |  |  |  |
| 6 | 0.9932 |  |  |  |
| 7 | 0.9932 |  |  |  |
| 8 | 0.9932 |  |  |  |
| 9 | 0.8356 |  |  |  |
| 10 | 0.8356 |  |  |  |
| 11 | 0.8356 |  |  |  |
| 12 | 0.8356 |  |  |  |
| 13 | 0.8356 |  |  |  |
| 14 | 0.8356 |  |  |  |
| 15 | 0.8219 |  |  |  |
| 16 | 0.8151 |  |  |  |
| 17 | 0.8082 |  |  |  |
| 18 | 0.8014 |  |  |  |
| 19 | 0.7877 |  |  |  |
| 20 | 0.7877 |  |  |  |
| 21 | 0.774 |  |  |  |
| 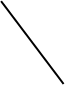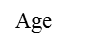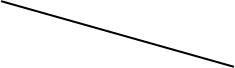   | 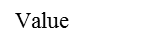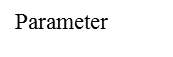 | | --- | | | *l_x_* | *f_x7_* | *m_x_* | *l_x_m_x_* |
|
| 22 | 0.774 |  |  |  |
| 23 | 0.7534 |  |  |  |
| 24 | 0.7534 |  |  |  |
| 25 | 0.7534 |  |  |  |
| 26 | 0.7534 |  |  |  |
| 27 | 0.7534 |  |  |  |
| 28 | 0.7534 |  |  |  |
| 29 | 0.7534 |  |  |  |
| 30 | 0.7534 |  |  |  |
| 31 | 0.7534 |  |  |  |
| 32 | 0.7534 |  |  |  |
| 33 | 0.7534 | 0 | 0 | 0 |
| 34 | 0.7466 | 1.1579 | 0.2018 | 0.1507 |
| 35 | 0.7466 | 6.5862 | 1.7523 | 1.3082 |
| 36 | 0.7466 | 17.9189 | 6.0826 | 4.5411 |
| 37 | 0.7466 | 22.2821 | 7.9725 | 5.9521 |
| 38 | 0.7466 | 24.2326 | 9.5596 | 7.137 |
| 39 | 0.7466 | 23.2955 | 9.4037 | 7.0205 |
| 40 | 0.7466 | 26.3958 | 11.6239 | 8.6781 |
| 41 | 0.7466 | 25.449 | 11.4404 | 8.5411 |
| 42 | 0.7466 | 29.02 | 13.3119 | 9.9384 |
| 43 | 0.7397 | 26.2653 | 11.9167 | 8.8151 |
| 44 | 0.7329 | 23.6122 | 10.8131 | 7.9247 |
| 45 | 0.7329 | 19.898 | 9.1121 | 6.6781 |
| 46 | 0.726 | 18.7143 | 8.6509 | 6.2808 |
| 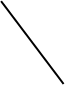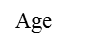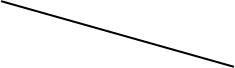   | 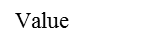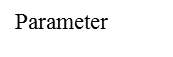 | | --- | | | *l_x_* | *f_x7_* | *m_x_* | *l_x_m_x_* |
|
| 47 | 0.6986 | 16.3125 | 7.6765 | 5.363 |
| 48 | 0.6849 | 15.6383 | 7.35 | 5.0342 |
| 49 | 0.6781 | 15 | 7.1212 | 4.8288 |
| 50 | 0.6644 | 12.6739 | 6.0103 | 3.9932 |
| 51 | 0.6575 | 10.8 | 5.0625 | 3.3288 |
| 52 | 0.6164 | 10.625 | 4.7222 | 2.911 |
| 53 | 0.6027 | 9.0263 | 3.8977 | 2.3493 |
| 54 | 0.5822 | 8.6486 | 3.7647 | 2.1918 |
| 55 | 0.5479 | 7.4167 | 3.3375 | 1.8288 |
| 56 | 0.5137 | 6.3235 | 2.8667 | 1.4726 |
| 57 | 0.4863 | 6.5 | 2.9296 | 1.4247 |
| 58 | 0.4589 | 5.0313 | 2.403 | 1.1027 |
| 59 | 0.4315 | 3.6667 | 1.746 | 0.7534 |
| 60 | 0.4041 | 2.4074 | 1.1017 | 0.4452 |
| 61 | 0.3699 | 1.6957 | 0.7222 | 0.2671 |
| 62 | 0.3356 | 1.2727 | 0.5714 | 0.1918 |
| 63 | 0.3219 | 1.5455 | 0.7234 | 0.2329 |
| 64 | 0.3014 | 1.85 | 0.8409 | 0.2534 |
| 65 | 0.274 | 0.3889 | 0.175 | 0.0479 |
| 66 | 0.2329 | 0.2667 | 0.1176 | 0.0274 |
| 67 | 0.1986 | 0 | 0 | 0 |
| 68 | 0.1644 | 0.3636 | 0.1667 | 0.0274 |
| 69 | 0.137 | 0 | 0 | 0 |
| 70 | 0.1301 | 0.125 | 0.0526 | 6.85E-03 |
| 71 | 0.1164 | 0 | 0 | 0 |
| 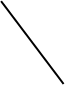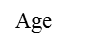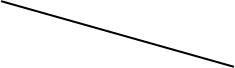   | 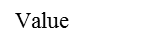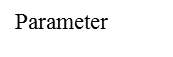 | | --- | | | *l_x_* | *f_x7_* | *m_x_* | *l_x_m_x_* |
|
| 72 | 0.1096 |  |  |  |
| 73 | 0.0822 |  |  |  |
| 74 | 0.0616 |  |  |  |
| 75 | 0.0342 |  |  |  |
| 76 | 0.0205 |  |  |  |
| 77 | 0.0137 |  |  |  |
| 78 | 6.85E-03 |  |  |  |
| 79 | 6.85E-03 |  |  |  |
| 80 | 0 |  |  |  |

**24℃**

| 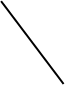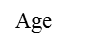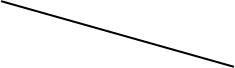   | 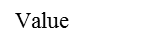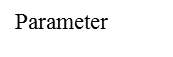 | | --- | | | *l_x_* | *f_x7_* | *m_x_* | *l_x_m_x_* |
| --- | --- | --- | --- | --- | --- |
|
| 0 | 1 |  |  |  |
| 1 | 1 |  |  |  |
| 2 | 1 |  |  |  |
| 3 | 1 |  |  |  |
| 4 | 1 |  |  |  |
| 5 | 1 |  |  |  |
| 6 | 0.9714 |  |  |  |
| 7 | 0.8214 |  |  |  |
| 8 | 0.8071 |  |  |  |
| 9 | 0.7786 |  |  |  |
| 10 | 0.7786 |  |  |  |
| 11 | 0.7786 |  |  |  |
| 12 | 0.7714 |  |  |  |
| 13 | 0.7714 |  |  |  |
| 14 | 0.7714 |  |  |  |
| 15 | 0.7714 |  |  |  |
| 16 | 0.7643 |  |  |  |
| 17 | 0.7643 |  |  |  |
| 18 | 0.7429 |  |  |  |
| 19 | 0.7429 | 0 | 0 | 0 |
| 20 | 0.7429 | 23.5625 | 3.625 | 2.6929 |
| 21 | 0.7357 | 28.8621 | 8.1262 | 5.9786 |
| 22 | 0.7357 | 37.4444 | 13.0874 | 9.6286 |
| 23 | 0.7357 | 33.9167 | 11.8544 | 8.7214 |
| 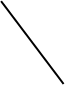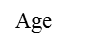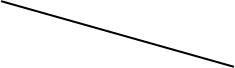   | 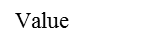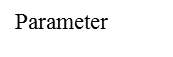 | | --- | | | *l_x_* | *f_x7_* | *m_x_* | *l_x_m_x_* |
|
| 24 | 0.7357 | 33.9459 | 12.1942 | 8.9714 |
| 25 | 0.7357 | 28.9211 | 10.6699 | 7.85 |
| 26 | 0.7357 | 26.9318 | 11.5049 | 8.4643 |
| 27 | 0.7 | 23.42 | 11.949 | 8.3643 |
| 28 | 0.6857 | 21.8909 | 12.5417 | 8.6 |
| 29 | 0.6857 | 22.1034 | 13.3542 | 9.1571 |
| 30 | 0.6857 | 16.931 | 10.2292 | 7.0143 |
| 31 | 0.6857 | 16.0172 | 9.6771 | 6.6357 |
| 32 | 0.6857 | 14.2586 | 8.6146 | 5.9071 |
| 33 | 0.6857 | 10.8966 | 6.5833 | 4.5143 |
| 34 | 0.6857 | 10.2414 | 6.1875 | 4.2429 |
| 35 | 0.6786 | 7.5439 | 4.5263 | 3.0714 |
| 36 | 0.6786 | 7.386 | 4.4316 | 3.0071 |
| 37 | 0.6643 | 5.8909 | 3.4839 | 2.3143 |
| 38 | 0.65 | 6.2075 | 3.6154 | 2.35 |
| 39 | 0.6286 | 4.82 | 2.7386 | 1.7214 |
| 40 | 0.6143 | 4.1042 | 2.2907 | 1.4071 |
| 41 | 0.6071 | 3.5106 | 1.9412 | 1.1786 |
| 42 | 0.5929 | 2.6889 | 1.4578 | 0.8643 |
| 43 | 0.5714 | 2 | 1.05 | 0.6 |
| 44 | 0.5714 | 1.4286 | 0.75 | 0.4286 |
| 45 | 0.5571 | 1.475 | 0.7564 | 0.4214 |
| 46 | 0.55 | 0.7436 | 0.3766 | 0.2071 |
| 47 | 0.5214 | 0.4 | 0.1918 | 0.1 |
| 48 | 0.5071 | 0.303 | 0.1408 | 0.0714 |
| 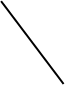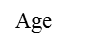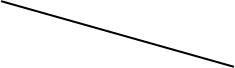   | 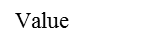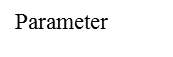 | | --- | | | *l_x_* | *f_x7_* | *m_x_* | *l_x_m_x_* |
|
| 49 | 0.4429 | 0.3704 | 0.1613 | 0.0714 |
| 50 | 0.4357 | 0.1154 | 0.0492 | 0.0214 |
| 51 | 0.4 | 0.0909 | 0.0357 | 0.0143 |
| 52 | 0.35 | 0 | 0 | 0 |
| 53 | 0.3143 |  |  |  |
| 54 | 0.2643 |  |  |  |
| 55 | 0.2357 |  |  |  |
| 56 | 0.1857 |  |  |  |
| 57 | 0.1714 |  |  |  |
| 58 | 0.1571 |  |  |  |
| 59 | 0.1071 |  |  |  |
| 60 | 0.0857 |  |  |  |
| 61 | 0.0714 |  |  |  |
| 62 | 0.0429 |  |  |  |
| 63 | 0.0286 |  |  |  |
| 64 | 0.0214 |  |  |  |
| 65 | 0.0143 |  |  |  |
| 66 | 0.0143 |  |  |  |
| 67 | 0.0143 |  |  |  |
| 68 | 7.14E-03 |  |  |  |
| 69 | 0 |  |  |  |

**27℃**

| 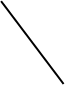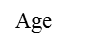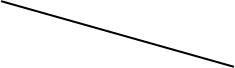   | 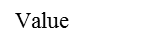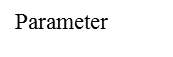 | | --- | | | *l_x_* | *f_x7_* | *m_x_* | *l_x_m_x_* |
| --- | --- | --- | --- | --- | --- |
|
| 0 | 1 |  |  |  |
| 1 | 1 |  |  |  |
| 2 | 1 |  |  |  |
| 3 | 1 |  |  |  |
| 4 | 1 |  |  |  |
| 5 | 1 |  |  |  |
| 6 | 0.9933 |  |  |  |
| 7 | 0.92 |  |  |  |
| 8 | 0.92 |  |  |  |
| 9 | 0.92 |  |  |  |
| 10 | 0.92 |  |  |  |
| 11 | 0.9 |  |  |  |
| 12 | 0.9 |  |  |  |
| 13 | 0.8867 |  |  |  |
| 14 | 0.8667 |  |  |  |
| 15 | 0.86 |  |  |  |
| 16 | 0.86 |  |  |  |
| 17 | 0.86 |  |  |  |
| 18 | 0.86 |  |  |  |
| 19 | 0.86 |  |  |  |
| 20 | 0.8533 | 0 | 0 | 0 |
| 21 | 0.8533 | 6.1667 | 0.2891 | 0.2467 |
| 22 | 0.8533 | 14.2857 | 3.9063 | 3.3333 |
| 23 | 0.8533 | 21.2373 | 9.7891 | 8.3533 |
| 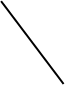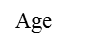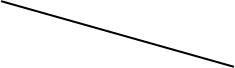   | 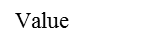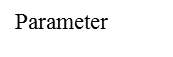 | | --- | | | *l_x_* | *f_x7_* | *m_x_* | *l_x_m_x_* |
|
| 24 | 0.8467 | 25.2308 | 12.9134 | 10.9333 |
| 25 | 0.8467 | 27.7123 | 15.9291 | 13.4867 |
| 26 | 0.8467 | 25.5676 | 14.8976 | 12.6133 |
| 27 | 0.8467 | 21.1757 | 12.3386 | 10.4467 |
| 28 | 0.8467 | 24.4865 | 14.2677 | 12.08 |
| 29 | 0.8467 | 23.6486 | 13.7795 | 11.6667 |
| 30 | 0.8467 | 18.7973 | 10.9528 | 9.2733 |
| 31 | 0.8467 | 21.4865 | 12.5197 | 10.6 |
| 32 | 0.8467 | 16.8919 | 9.8425 | 8.3333 |
| 33 | 0.8467 | 15.2973 | 8.9134 | 7.5467 |
| 34 | 0.8467 | 13.5946 | 7.9213 | 6.7067 |
| 35 | 0.8333 | 11.1644 | 6.52 | 5.4333 |
| 36 | 0.8333 | 7.8219 | 4.568 | 3.8067 |
| 37 | 0.82 | 5.9726 | 3.5447 | 2.9067 |
| 38 | 0.8 | 4.7808 | 2.9083 | 2.3267 |
| 39 | 0.7733 | 3.6479 | 2.2328 | 1.7267 |
| 40 | 0.7667 | 3.4429 | 2.0957 | 1.6067 |
| 41 | 0.7533 | 2.5294 | 1.5221 | 1.1467 |
| 42 | 0.7333 | 1.8154 | 1.0727 | 0.7867 |
| 43 | 0.72 | 1.2969 | 0.7685 | 0.5533 |
| 44 | 0.7 | 1.5238 | 0.9143 | 0.64 |
| 45 | 0.6867 | 0.7419 | 0.4466 | 0.3067 |
| 46 | 0.6733 | 1.0328 | 0.6238 | 0.42 |
| 47 | 0.6533 | 0.431 | 0.2551 | 0.1667 |
| 48 | 0.6333 | 0.2 | 0.1158 | 0.0733 |
| 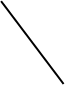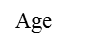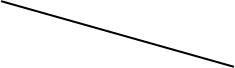   | 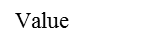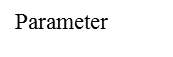 | | --- | | | *l_x_* | *f_x7_* | *m_x_* | *l_x_m_x_* |
|
| 49 | 0.6 | 0.0769 | 0.0444 | 0.0267 |
| 50 | 0.5733 | 0.0625 | 0.0349 | 0.02 |
| 51 | 0.5467 | 0.0227 | 0.0122 | 6.67E-03 |
| 52 | 0.52 | 0 | 0 | 0 |
| 53 | 0.4667 | 0.0294 | 0.0143 | 6.67E-03 |
| 54 | 0.4533 | 0.0313 | 0.0147 | 6.67E-03 |
| 55 | 0.4 | 0.037 | 0.0167 | 6.67E-03 |
| 56 | 0.3867 | 0 | 0 | 0 |
| 57 | 0.36 |  |  |  |
| 58 | 0.3133 |  |  |  |
| 59 | 0.3067 |  |  |  |
| 60 | 0.2533 |  |  |  |
| 61 | 0.22 |  |  |  |
| 62 | 0.2 |  |  |  |
| 63 | 0.1867 |  |  |  |
| 64 | 0.1667 |  |  |  |
| 65 | 0.1533 |  |  |  |
| 66 | 0.1333 |  |  |  |
| 67 | 0.1267 |  |  |  |
| 68 | 0.1067 |  |  |  |
| 69 | 0.0933 |  |  |  |
| 70 | 0.08 |  |  |  |
| 71 | 0.0667 |  |  |  |
| 72 | 0.04 |  |  |  |
| 73 | 0.0333 |  |  |  |
| 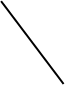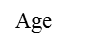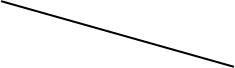   | 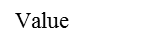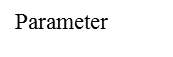 | | --- | | | *l_x_* | *f_x7_* | *m_x_* | *l_x_m_x_* |
|
| 74 | 0.0267 |  |  |  |
| 75 | 0.0267 |  |  |  |
| 76 | 0.0267 |  |  |  |
| 77 | 0.02 |  |  |  |
| 78 | 0.02 |  |  |  |
| 79 | 0.02 |  |  |  |
| 80 | 0.02 |  |  |  |
| 81 | 0.02 |  |  |  |
| 82 | 0.0133 |  |  |  |
| 83 | 6.67E-03 |  |  |  |
| 84 | 6.67E-03 |  |  |  |
| 85 | 6.67E-03 |  |  |  |
| 86 | 6.67E-03 |  |  |  |
| 87 | 6.67E-03 |  |  |  |
| 88 | 6.67E-03 |  |  |  |
| 89 | 6.67E-03 |  |  |  |
| 90 | 0 |  |  |  |
|  |  |  |  |  |

**30℃**

| 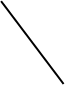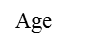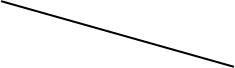   | 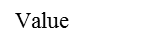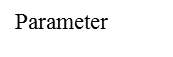 | | --- | | | *l_x_* | *f_x7_* | *m_x_* | *l_x_m_x_* |
| --- | --- | --- | --- | --- | --- |
|
| 0 | 1 |  |  |  |
| 1 | 1 |  |  |  |
| 2 | 1 |  |  |  |
| 3 | 1 |  |  |  |
| 4 | 0.9133 |  |  |  |
| 5 | 0.9067 |  |  |  |
| 6 | 0.9 |  |  |  |
| 7 | 0.9 |  |  |  |
| 8 | 0.9 |  |  |  |
| 9 | 0.8933 |  |  |  |
| 10 | 0.8667 |  |  |  |
| 11 | 0.8533 |  |  |  |
| 12 | 0.8467 |  |  |  |
| 13 | 0.8267 |  |  |  |
| 14 | 0.8133 |  |  |  |
| 15 | 0.7933 |  |  |  |
| 16 | 0.78 |  |  |  |
| 17 | 0.78 | 0 | 0 | 0 |
| 18 | 0.7733 | 12.2 | 3.1552 | 2.44 |
| 19 | 0.76 | 37.7174 | 15.2193 | 11.5667 |
| 20 | 0.7333 | 48.4167 | 21.1273 | 15.4933 |
| 21 | 0.7333 | 45.02 | 20.4636 | 15.0067 |
| 22 | 0.7333 | 42.08 | 19.1273 | 14.0267 |
| 23 | 0.7267 | 34.88 | 16 | 11.6267 |
| 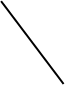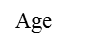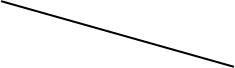   | 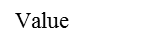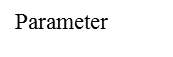 | | --- | | | *l_x_* | *f_x7_* | *m_x_* | *l_x_m_x_* |
|
| 24 | 0.7133 | 26.8367 | 12.2897 | 8.7667 |
| 25 | 0.7133 | 21.7959 | 9.9813 | 7.12 |
| 26 | 0.7133 | 15.0612 | 6.8972 | 4.92 |
| 27 | 0.7133 | 13.6939 | 6.271 | 4.4733 |
| 28 | 0.7133 | 10.7143 | 4.9065 | 3.5 |
| 29 | 0.7067 | 8.0612 | 3.7264 | 2.6333 |
| 30 | 0.6867 | 6.0638 | 2.767 | 1.9 |
| 31 | 0.6467 | 4.6364 | 2.1031 | 1.36 |
| 32 | 0.5867 | 3.6341 | 1.6932 | 0.9933 |
| 33 | 0.5533 | 3.3784 | 1.506 | 0.8333 |
| 34 | 0.52 | 1.6471 | 0.7179 | 0.3733 |
| 35 | 0.44 | 0.3333 | 0.1364 | 0.06 |
| 36 | 0.36 | 0 | 0 | 0 |
| 37 | 0.2867 |  |  |  |
| 38 | 0.2333 |  |  |  |
| 39 | 0.18 |  |  |  |
| 40 | 0.1333 |  |  |  |
| 41 | 0.0867 |  |  |  |
| 42 | 0.04 |  |  |  |
| 43 | 0.0267 |  |  |  |
| 44 | 0.02 |  |  |  |
| 45 | 6.67E-03 |  |  |  |
| 46 | 6.67E-03 |  |  |  |
| 47 | 6.67E-03 |  |  |  |
| 48 | 6.67E-03 |  |  |  |
| 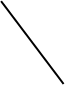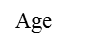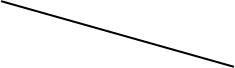   | 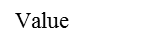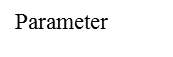 | | --- | | | *l_x_* | *f_x7_* | *m_x_* | *l_x_m_x_* |
|
| 49 | 6.67E-03 |  |  |  |
| 50 | 6.67E-03 |  |  |  |
| 51 | 6.67E-03 |  |  |  |
| 52 | 6.67E-03 |  |  |  |
| 53 | 6.67E-03 |  |  |  |
| 54 | 6.67E-03 |  |  |  |
| 55 | 6.67E-03 |  |  |  |
| 56 | 6.67E-03 |  |  |  |
| 57 | 6.67E-03 |  |  |  |
| 58 | 6.67E-03 |  |  |  |
| 59 | 0 |  |  |  |

**33℃**

| 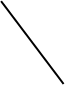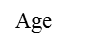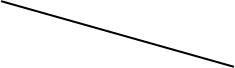   | 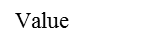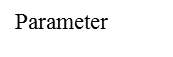 | | --- | | | *l_x_* | *f_x7_* | *m_x_* | *l_x_m_x_* |
| --- | --- | --- | --- | --- | --- |
|
| 0 | 1 |  |  |  |
| 1 | 1 |  |  |  |
| 2 | 1 |  |  |  |
| 3 | 1 |  |  |  |
| 4 | 0.9732 |  |  |  |
| 5 | 0.8188 |  |  |  |
| 6 | 0.7987 |  |  |  |
| 7 | 0.7987 |  |  |  |
| 8 | 0.7852 |  |  |  |
| 9 | 0.7785 |  |  |  |
| 10 | 0.7785 |  |  |  |
| 11 | 0.7718 |  |  |  |
| 12 | 0.7651 |  |  |  |
| 13 | 0.7651 |  |  |  |
| 14 | 0.7651 | 0 | 0 | 0 |
| 15 | 0.7517 | 0.4286 | 0.1339 | 0.1007 |
| 16 | 0.7383 | 0.3333 | 0.1545 | 0.1141 |
| 17 | 0.7315 | 0.7963 | 0.3945 | 0.2886 |
| 18 | 0.7315 | 0.8364 | 0.422 | 0.3087 |
| 19 | 0.7181 | 2.2182 | 1.1402 | 0.8188 |
| 20 | 0.7181 | 1.5536 | 0.8131 | 0.5839 |
| 21 | 0.7181 | 1.4286 | 0.7477 | 0.5369 |
| 22 | 0.7181 | 1.7321 | 0.9065 | 0.651 |
| 23 | 0.7114 | 1.5714 | 0.8302 | 0.5906 |
| 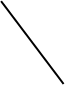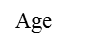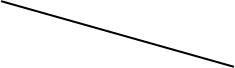   | 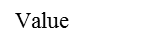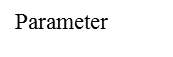 | | --- | | | *l_x_* | *f_x7_* | *m_x_* | *l_x_m_x_* |
|
| 24 | 0.7114 | 2.4821 | 1.3113 | 0.9329 |
| 25 | 0.7047 | 2.4909 | 1.3048 | 0.9195 |
| 26 | 0.6913 | 1.9623 | 1.0097 | 0.698 |
| 27 | 0.6913 | 1.1887 | 0.6117 | 0.4228 |
| 28 | 0.6913 | 0.6415 | 0.3301 | 0.2282 |
| 29 | 0.6779 | 1.0392 | 0.5248 | 0.3557 |
| 30 | 0.6779 | 1.0392 | 0.5248 | 0.3557 |
| 31 | 0.6644 | 0.3922 | 0.202 | 0.1342 |
| 32 | 0.6577 | 0.3 | 0.1531 | 0.1007 |
| 33 | 0.6309 | 0.3333 | 0.1702 | 0.1074 |
| 34 | 0.5839 | 0 | 0 | 0 |
| 35 | 0.5503 | 0.0698 | 0.0366 | 0.0201 |
| 36 | 0.5436 | 0.1395 | 0.0741 | 0.0403 |
| 37 | 0.5235 | 0.1667 | 0.0897 | 0.047 |
| 38 | 0.5101 | 0.0714 | 0.0395 | 0.0201 |
| 39 | 0.4698 | 0.0256 | 0.0143 | 6.71E-03 |
| 40 | 0.4295 | 0 | 0 | 0 |
| 41 | 0.3893 | 0.1667 | 0.1034 | 0.0403 |
| 42 | 0.3557 | 0 | 0 | 0 |
| 43 | 0.3289 |  |  |  |
| 44 | 0.3087 |  |  |  |
| 45 | 0.2819 |  |  |  |
| 46 | 0.2752 |  |  |  |
| 47 | 0.2416 |  |  |  |
| 48 | 0.1946 |  |  |  |
| 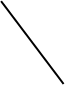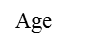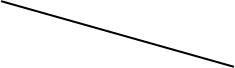   | 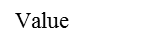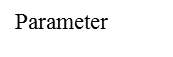 | | --- | | | *l_x_* | *f_x7_* | *m_x_* | *l_x_m_x_* |
|
| 49 | 0.1745 |  |  |  |
| 50 | 0.1342 |  |  |  |
| 51 | 0.1074 |  |  |  |
| 52 | 0.0738 |  |  |  |
| 53 | 0.047 |  |  |  |
| 54 | 0.0403 |  |  |  |
| 55 | 0.0134 |  |  |  |
| 56 | 6.71E-03 |  |  |  |
| 57 | 6.71E-03 |  |  |  |
| 58 | 6.71E-03 |  |  |  |
| 59 | 0 |  |  |  |
